# ILF3 regulates erythroid differentiation: A single-cell transcriptomic analysis and cellular experiment

**DOI:** 10.1371/journal.pone.0355975

**Published:** 2026-09-03

**Authors:** Xiaojing Wu, Meng Lu, Wenguang Jia, Weixiong Lin, Yunyan He

**Affiliations:** 1 Department of Pediatrics, The First Affiliated Hospital of Guangxi Medical University, Nanning, China; 2 Graduate School, Guangxi Medical University, Nanning, China; 3 NHC Key Laboratory of Thalassemia Medicine, Chinese Academy of Medical Sciences, Nanning, China; 4 Guangxi Key Laboratory of Thalassemia Research, Nanning, China; Chuo University, JAPAN

## Abstract

**Background:**

Despite extensive knowledge of erythropoiesis, the role of interleukin enhancer binding factor 3 (ILF3) in β-thalassemia remains unclear. The aim of this study was to investigate the expression pattern and functional role of ILF3 during erythroid differentiation in β-thalassemia.

**Methods:**

(1) Single-cell transcriptomic profiles were constructed based on the GSE133181 dataset (3 healthy controls and 3 β-thalassemia patients). The differential expression of ILF3, transcriptional programs of erythroid subsets, pathway activity, and differentiation trajectory were analyzed. (2) ILF3 was knocked out in K562 cells using sgRNA. Cell proliferation was detected by CCK-8 assay, and apoptosis was measured by Annexin V-APC single staining.

**Results:**

(1) ILF3 was downregulated in the β-thalassemia group, and the proportion of erythroid cells was significantly enriched in ILF3^+^ cells. (2) ILF3^+^ erythroid cells highly expressed maturation-related genes and were enriched in cell cycle and metabolic synthesis pathways, while ILF3^−^ cells highly expressed stress-related genes and activated inflammatory stress pathways. (3) Trajectory analysis suggested early arrest of erythroid differentiation in β-thalassemia, with consistently low ILF3 expression throughout the disease group. (4) Knockout of ILF3 promoted apoptosis in K562 cells.

**Conclusion:**

ILF3 is downregulated in β-thalassemia, and its deficiency may contribute to erythroid differentiation arrest and ineffective hematopoiesis by inducing apoptosis and potentially enhancing inflammatory stress.

## Introduction

Beta-thalassemia (β-thalassemia) is a common inherited hemoglobinopathy characterized by reduced or absent synthesis of the β-globin chain, leading to the accumulation of free α-globin chains that form toxic aggregates. This pathological process results in ineffective erythropoiesis, manifested by impaired differentiation and maturation of erythroid cells and increased apoptosis [1–3]. Current clinical treatments mainly include blood transfusion, iron chelation, and hematopoietic stem cell transplantation. However, these approaches are limited by numerous complications and difficulties in donor matching, highlighting an urgent need to explore novel intervention targets at the molecular mechanism level.

The regulation of erythroid differentiation is a complex, multi-stage and multi-level process, coordinately controlled by various factors including transcription factors, non-coding RNAs, and signaling pathways. Among these, transcription factors play crucial roles in erythroid differentiation and maturation [4–6]. Studies have shown that Krüppel-like transcription factors KLF1 and KLF2 are essential for embryonic erythropoiesis, as they cooperatively regulate the maturation of embryonic erythroid progenitor cells by modulating multiple homeostasis-related genes [7]. In addition, GATA1 and GATA2, members of the GATA transcription factor family, also exert important functions during erythroid differentiation. GATA1 ensures the survival and terminal differentiation of erythroid, megakaryocytic, and eosinophilic precursors, whereas GATA2 regulates the proliferation and maintenance of hematopoietic stem and progenitor cells. The dynamic switch between GATA1 and GATA2 guarantees the stage-specific gene expression during erythroid differentiation [8]. ILF3 is a multifunctional RNA-binding protein involved in transcription regulation, mRNA stability, and translation [9]. It plays a critical role in cell proliferation and differentiation [10]. A study has shown that ILF3 acts as a transcription factor that promotes proliferation via hierarchical regulation in K562 erythroleukemia cells [11]. Our previous work also suggested that ILF3 may participate in regulating γ-globin gene expression [12]. However, its expression pattern and function in normal erythroid differentiation and the pathogenesis of β-thalassemia remain unclear.

Single-cell RNA sequencing provides unprecedented resolution for dissecting the spatiotemporal heterogeneity of gene expression in complex cell populations and delineating cell differentiation trajectories. Through single-cell transcriptomic analysis of hematopoietic stem cells from β-thalassemia patients, researchers have identified diverse gene expression patterns associated with erythroid differentiation, offering new insights into understanding the phenotypic heterogeneity of β-thalassemia [13]. In this study, we integrated public single-cell datasets to systematically analyze the expression profile of ILF3 in hematopoietic cells of β-thalassemia patients. We further performed functional validation using the K562 cell line. This study aims to clarify the role of ILF3 in erythroid differentiation and its underlying mechanism in ineffective hematopoiesis in β-thalassemia, thereby providing new strategies for molecular targeted therapy of the disease.

## Materials and methods

### Acquisition of single-cell RNA sequencing (scRNA-seq) data

The single-cell RNA sequencing (scRNA-seq) dataset was obtained from the Gene Expression Omnibus (GEO) database under accession number GSE133181, which includes bone marrow CD34^+^ cells collected from healthy donors and patients with hemoglobinopathies [14]. Downstream analyses were performed using the Seurat R package [15]. Quality control was applied to exclude low-quality cells based on the following criteria: cells with fewer than 1,000 unique molecular identifiers (UMIs), fewer than 500 detected genes, or mitochondrial gene content exceeding 6% were removed. Data standardization and correction were conducted by NormalizeData, which selected 2000 highly variable genes for subsequent analysis. Removal of batch effect was implemented using Harmony [16]. The subsequent usage of dimensionality reduction methods included UMAP, TSNE, and clustering algorithm Louvain, all from Seurat. Differentially expressed genes (DEGs) between clusters or predefined cell groups were identified using the FindAllMarkers function. Genes with a p-value < 0.05, an absolute log_2_ fold change (|log_2_FC|) > 0.25, and an expression proportion > 0.1 were considered statistically significant.

### Cell type annotation

Cell type annotation was performed based on classic marker genes. Erythroid cells were annotated by HBB, TMEM14C, and BLVRB. Hematopoietic stem and progenitor cells (HSPCs) were annotated by AVP, HOPX, and SPINK2. Granulocyte-macrophage progenitors (GMPs) were annotated by LYZ, MPO, and ELANE. Plasmacytoid dendritic cells (pDCs) were annotated by LGMN, IL3RA, and IRF7. And Pre-B cells were annotated by DNTT, IGHM, and VPREB1. The results of cell annotation were visualized using UMAP plots, and the expression patterns of the aforementioned marker genes were displayed via dot plots.

### Comparison of ILF3 expression levels

ILF3 expression levels were extracted from the normalized RNA dataset of the Seurat object. Violin plots were generated using the ggplot2 package (version 3.5.1) in R to compare the differential expression of ILF3 between β-thalassemia samples and healthy control samples. A two-tailed Wilcoxon rank-sum test (Mann–Whitney U test) was used to evaluate statistical differences between groups, with P < 0.05 considered statistically significant.

### Cell grouping and composition analysis based on ILF3 expression

Cells were stratified into ILF3-positive (ILF3^+^) and ILF3-negative (ILF3^−^) groups using the median ILF3 expression level across all cells as the threshold. Stacked bar charts illustrate the proportional distribution of pre-annotated cell types within each group.

### Transcriptional program differences in erythroid subgroups stratified by ILF3 expression

To explore the differences in erythroid-associated transcriptional programs between ILF3^+^ and ILF3^−^ erythroid cells, key erythroid-related genes were selected according to previous studies, including HBB, HBA1, HBA2, KLF1, ALAS2, BCL11A, ZBTB7A, MYB, and DNMT3A. Gene expression levels were visualized using violin plots or dot plots. A two-tailed Wilcoxon rank-sum test was used to evaluate statistical differences between groups, with P < 0.05 considered statistically significant.

### Cell trajectory and pseudotime analysis

Cell trajectory inference and pseudotime analysis were performed using the Monocle3 (R package, version 1.4.26) [17]. Hematopoietic stem and progenitor cells and erythroid subsets were extracted from the integrated Seurat object and converted into the Monocle3 cell data set (CDS) format. UMAP dimensionality reduction and cell trajectory construction were performed based on the highly variable genes identified by Seurat. Hematopoietic stem and progenitor cells were set as the starting cell population for pseudotime ordering. Pseudotime-dependent differentially expressed genes were screened with a false discovery rate (FDR) < 0.01 as the threshold. Pseudotime distribution and dynamic expression patterns of representative genes (such as ILF3) were visualized using ggplot2.

### Integrated functional pathway activity analysis using multiple algorithms

To evaluate the differences in functional pathway activity between ILF3^+^ and ILF3^−^ erythroid cells, the MSigDB Hallmark gene set (version 7.5.1) [18] was used as the reference database. Four independent single-cell level algorithms were applied to calculate pathway activity scores: AUCell (version 1.18.0) [19], UCell (version 2.6.0) [20], singscore (version 1.16.0) [21], and single-sample gene set enrichment analysis (ssGSEA) implemented by the GSVA R package (version 1.44.2) [22].

### Cell culture

Human chronic myeloid leukemia cells (K562) were purchased from the Stem Cell Bank of the Chinese Academy of Sciences (Shanghai, China). K562 cells were cultured in Roswell Park Memorial Institute (RPMI) 1640 medium (Sigma-Aldrich, USA) supplemented with 100 IU/mL penicillin (Solarbio, China), 100 μg/mL streptomycin (Solarbio, China), and 10% fetal bovine serum (South American origin, Gibco, USA). Cells were maintained at 37°C in a 5% CO_2_ incubator.

### Lentiviral vector construction and infection

ILF3, also known as NF90, has a Gene ID of 3609 and GenBank accession number NM_012218. The CRISPR/Cas9-mediated ILF3 knockout (ILF3-KO) lentiviral vector (LV-ILF3-sgRNA (09814−1)) and the negative control virus CON244 (NC) were constructed by Shanghai GeneChem Co., Ltd. The specificity and correctness of the sgRNA sequences targeting *ILF3* were confirmed by Sanger sequencing prior to lentiviral packaging (S1 File). K562 cells were transfected with the above viruses according to the manufacturer’s instructions at a multiplicity of infection (MOI) of 20. Untreated K562 cells were set as the blank control (CON). Cells were monitored to ensure good viability without significant cell death, maintaining comparable conditions across all groups. Approximately 72 hours post-infection, green fluorescent protein (GFP) expression was visualized using a fluorescence microscope. The percentage of GFP-positive cells, representing the infection efficiency, was assessed. Once the infection rate reached approximately 80%, the cells were deemed suitable for downstream functional assays.

### Cell proliferation and apoptosis assays

To determine the effect of ILF3 knockout on K562 cell proliferation, cell viability was measured using the Cell Counting Kit-8 (CCK-8, Sigma, USA) according to the manufacturer’s protocol. K562 cells were seeded into 96-well plates at a density of approximately 2,000 cells per well in 100 μL of culture medium, with three replicates per group. Cell viability was assessed daily for 5 days using the Cell Counting Kit-8 (CCK-8) assay. Briefly, 10 μL of CCK-8 solution was added to each well 2–4 hours before the end of the incubation period without replacing the culture medium. The plates were then gently shaken for 2–5 minutes, and the optical density (OD) was measured at 450 nm using a microplate reader.

Meanwhile, apoptosis of K562 cells was analyzed by flow cytometry using the Annexin V-APC Apoptosis Detection Kit (eBioscience, San Diego, CA, USA) following the manufacturer’s instructions. K562 cells were seeded into 6-well plates (2 mL/well) and cultured for 5 days until reaching approximately 85% confluence. Cells were harvested by centrifugation at 1,300 rpm for 5 minutes and washed with pre-chilled D-Hanks solution. The cell pellets were then resuspended in 500 μL of 1 × binding buffer and centrifuged again at 1,300 rpm for 3 minutes. Subsequently, cells were resuspended in 200 μL of 1 × binding buffer and stained with 10 μL of Annexin V-APC at room temperature in the dark for 15 minutes. Finally, 400–800 μL of 1 × binding buffer was added to the samples, and the apoptosis rate was analyzed using a flow cytometer. All experiments were performed in triplicate.

### Statistical analysis

SPSS 26.0 software was used for statistical analysis. Normally distributed experimental data were expressed as mean ± standard deviation (mean ± SD), while non-normally distributed data were expressed as median and interquartile range. For normally distributed data, the t-test was used for comparison between two groups, and one-way analysis of variance (ANOVA) was used for comparison among multiple groups. For non-normally distributed data, the Mann Whitney U test was used for comparison between two groups, and the Kruskal Wallis H test was used for comparison among multiple groups. A P-value < 0.05 was considered statistically significant.

## Results

### Construction of single-cell atlas and cell type annotation

**Fig 1:**
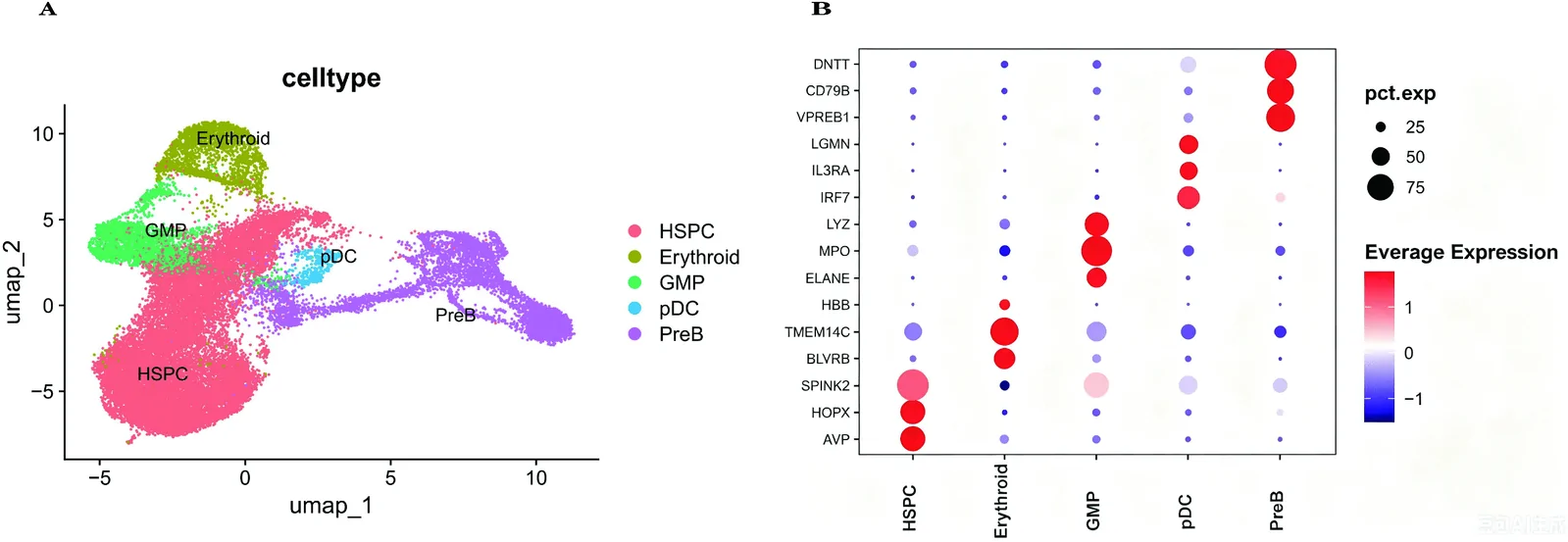
Single-cell atlas and cell type annotation of integrated GSE133181 dataset. (A) UMAP plot showing five major cell clusters: HSPC, Erythroid, GMP, pDC, and Pre-B cells, colored by identity. Data from healthy controls (n = 3) and β-thalassemia patients (n = 3) were integrated and batch-corrected. (B) Dot plot of canonical marker genes across clusters. Dot size = % expressing cells; color = average expression level (red: high, purple: low). Markers: AVP/HOPX/SPINK2 (HSPC), TMEM14C/BLVRB/HBB (Erythroid), MPO/ELANE/LYZ (GMP), IRF7/IL3RA/LGMN (pDC), DNTT/CD79B/VPREB1 (Pre-B).

We integrated the GSE133181 dataset (n = 3 for healthy controls, n = 3 for β-thalassemia). After normalization, batch effect correction, dimensionality reduction, and clustering analysis, a high-quality single-cell transcriptomic atlas was constructed. Based on classic marker genes, we annotated HSPC, GMP, pDC, pre-B cells, and erythroid cells. UMAP visualization showed distinct distribution of each subset, with a continuous differentiation axis formed between HSPC and erythroid cells, providing a structural basis for subsequent trajectory analysis (Fig 1).

### ILF3 was overall downregulated in β-thalassemia

**Fig 2:**
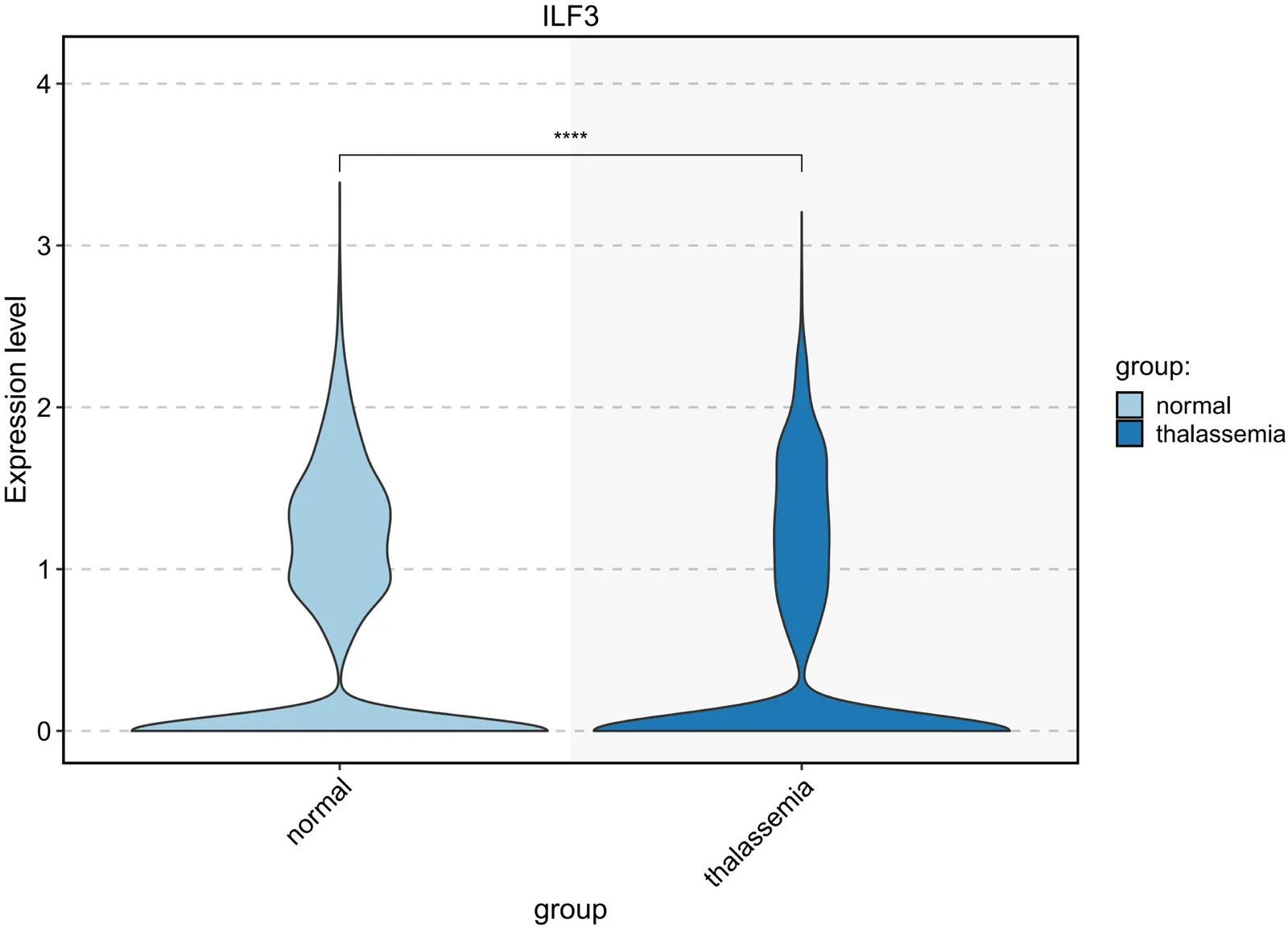
Comparison of ILF3 expression levels between normal and β-thalassemia patients. Violin plot showing significantly reduced ILF3 expression in β-thalassemia samples (n = 3) compared to normal group (n = 3; *p* < 0.0001). Distribution width indicates cell/sample density; center line = median.

Comparison at the sample level revealed that the overall expression level of ILF3 was significantly lower in the β-thalassemia group than in the healthy control group (violin plot, p < 0.05), suggesting systematic downregulation of ILF3 under disease conditions (Fig 2).

### Differences in cell composition based on ILF3 expression status

**Fig 3:**
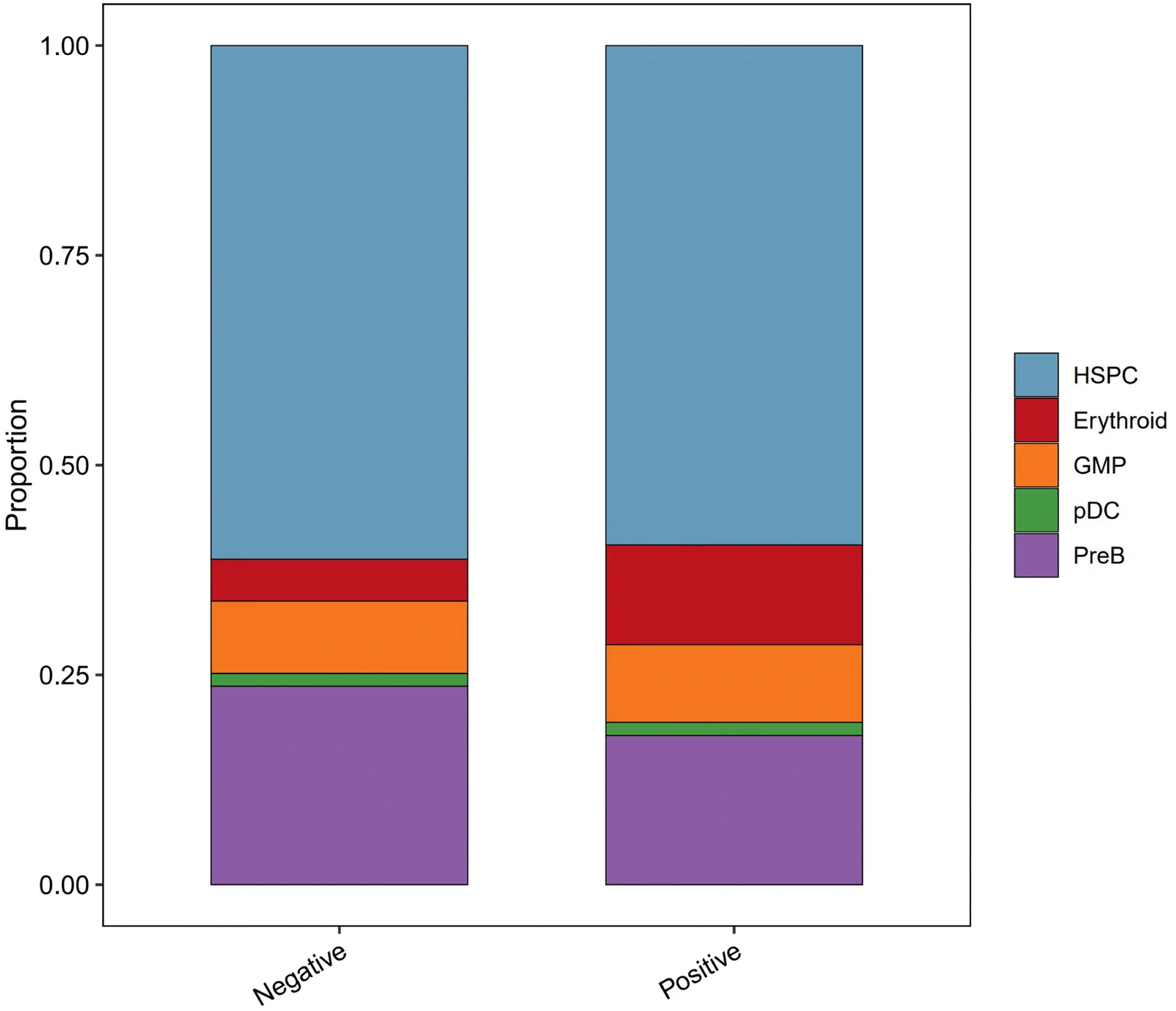
Lineage distribution in cells with or without ILF3 expression. Stacked bar plot showing cell type proportions in ILF3-negative vs. ILF3-positive populations. Erythroid cells are markedly enriched in the ILF3-positive group, while other lineages (HSPC, GMP, pDC, PreB) show minimal shifts.

All cells were divided into ILF3 positive (ILF3^+^) and ILF3 negative (ILF3^−^) groups according to ILF3 expression, and the proportion of each cell type in the two groups was analyzed. The results showed that the annotated erythroid cells were significantly enriched in the ILF3^+^ population, while no obvious distribution differences were observed for other cell types between the positive and negative groups, suggesting a specific association between ILF3 expression and erythroid differentiation (Fig 3).

### Transcriptional program differences within the erythroid population

**Fig 4:**
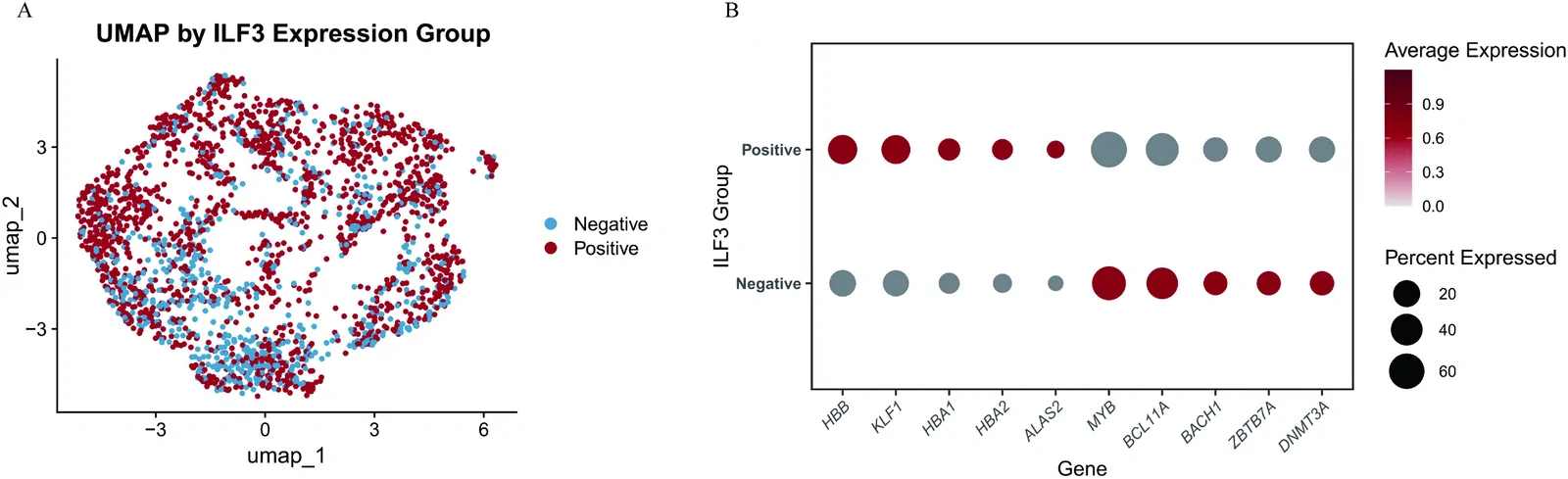
Characterization of cell distribution and gene expression profiles by ILF3 status. (A) UMAP plot colored by ILF3 expression status (Negative = blue, Positive = red). (B) Dot plot comparing gene expression between ILF3-positive and -negative erythroid cells. In ILF3-positive cells, maturation genes (HBB, HBA1/2, KLF1, ALAS2) are highly expressed; in ILF3-negative cells, stress/inhibitory factors (MYB, BCL11A, ZBTB7A, DNMT3A, BACH1) dominate.

Within the erythroid population, ILF3^+^ erythroid cells exhibited significantly elevated expression of key erythroid maturation genes (HBB, HBA1, HBA2), a critical maturation regulator (KLF1), and a heme synthesis gene (ALAS2), indicating that this population represents metabolically active erythroid cells at the mature stage. In contrast, ILF3^−^ erythroid cells showed significantly upregulated expression of stress related genes (BACH1), fetal hemoglobin repressors (BCL11A, ZBTB7A), and transcriptional repressors (MYB, DNMT3A), implying a potential state of developmental arrest or stress (Fig 4). These results indicate that ILF3 may maintain the homeostatic development of erythroid cells by activating erythroid maturation genes and suppressing stress related transcriptional programs.

### Differentiation trajectory reveals early erythroid arrest in β-thalassemia

**Fig 5:**
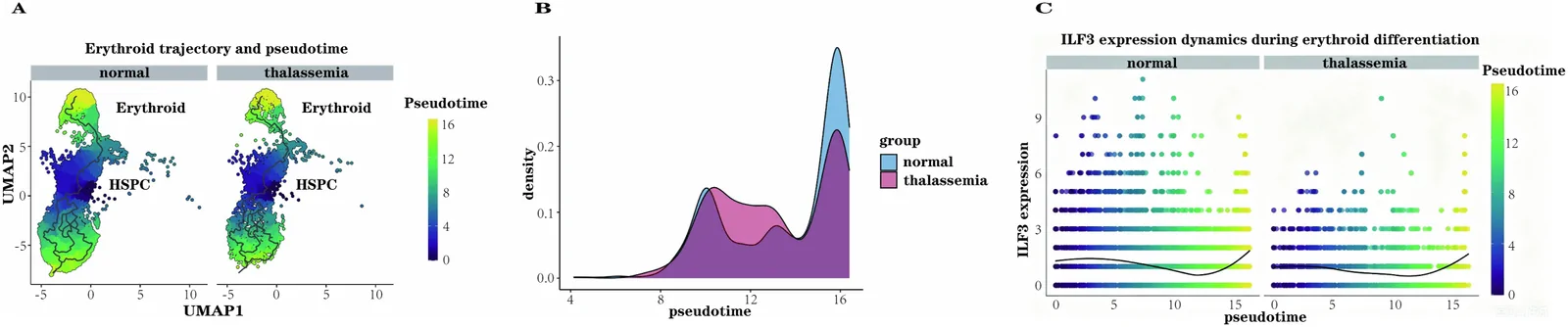
Erythroid differentiation trajectories and ILF3 dynamics in normal vs. β-thalassemia. (A) UMAP plots showing inferred differentiation trajectories for normal (left) and β-thalassemia (right) cells. Cells are colored by pseudotime (0 = start, 16 = end), with gray lines indicating developmental paths from HSPCs to erythroid cells. (B) Pseudotime density distributions comparing cell abundance. The β-thalassemia group (red) shows a distinct accumulation of cells at early stages (pseudotime ~8–12), whereas the normal group (blue) peaks at later stages. (C) Scatter plots of single-cell ILF3 expression against pseudotime. Dots represent individual cells (colored by pseudotime); solid black lines show smoothed expression trends. The β-thalassemia group exhibits a flatter expression trajectory during mid-differentiation compared to normal.

Differentiation trajectories from HSPCs to the erythroid lineage were reconstructed using Monocle3. Erythroid cells in the healthy group were mainly distributed at high pseudotime, representing the late differentiation stage, whereas those in the β-thalassemia group were significantly enriched at low pseudotime, corresponding to the early differentiation stage. Dynamic changes in ILF3 expression along pseudotime showed that ILF3 expression increased with differentiation progression in both groups, but was consistently higher in the healthy group and further elevated at the terminal stage, while it remained low throughout the thalassemia group. These results suggest that low ILF3 expression coincides with erythroid differentiation arrest in thalassemia samples (Fig 5).

### Differential functional pathways associated with ILF3

**Fig 6:**
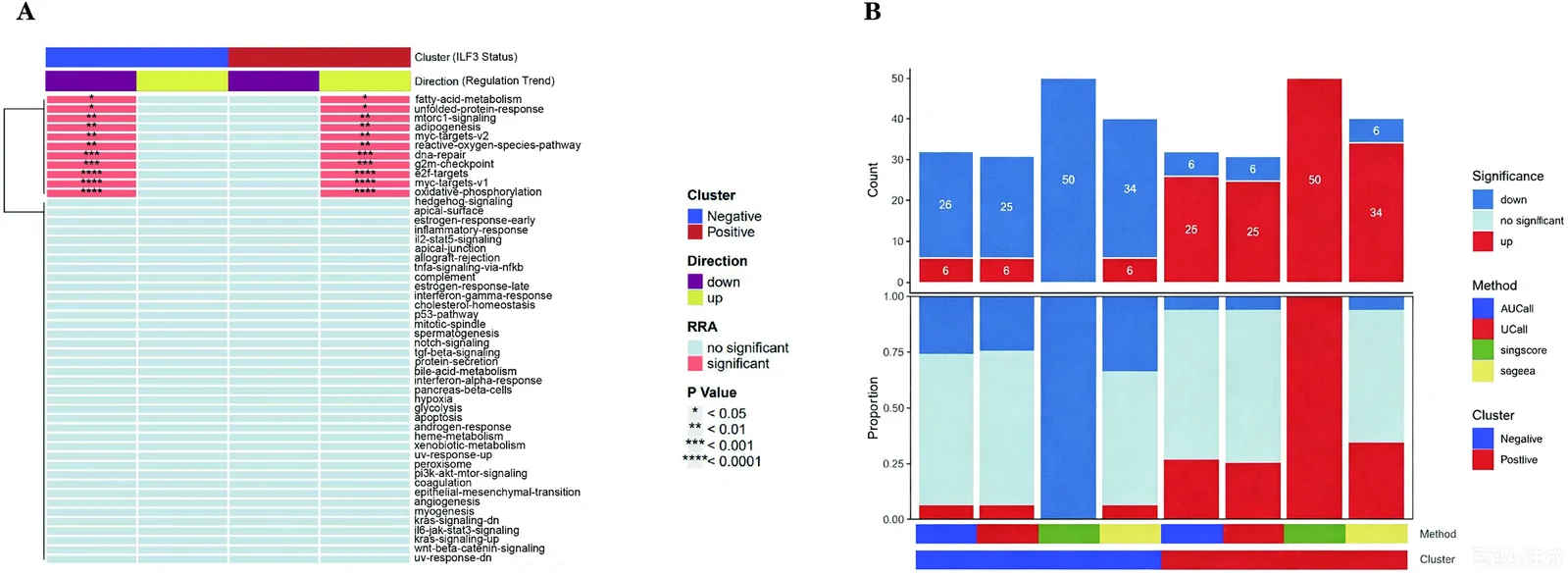
Pathway activity profiling of ILF3-positive and ILF3-negative erythroid cells using integrated single-cell scoring methods. (A) Heatmap of MSigDB Hallmark pathway enrichment scores. Top row: ILF3 status (Blue = ILF3^+^ erythroid cells, Red = ILF3^−^ erythroid cells). Second row: Pathway regulation trend (Purple = Downregulated, Yellow = Upregulated).

Based on the MSigDB Hallmark collection, we performed single-cell pathway scoring in ILF3-positive and ILF3-negative erythroid cells, and integrated results from the four algorithms (AUCell, UCell, singscore, and ssGSEA) using RRA. As shown in Fig 6A, multiple pathways were significantly upregulated in ILF3-positive cells, including G2M checkpoint, E2F targets, MYC targets, DNA repair, oxidative phosphorylation, fatty acid metabolism, and heme metabolism. By contrast, Interferon α/γ response, TNF-α/NF-κB signaling, IL6–JAK–STAT3 signaling, complement, allograft rejection and inflammatory response were markedly activated in ILF3-negative cells. All four methods yielded consistent trends of pathway regulation across the two cell populations (Fig 6B), which verified the reliability of our findings.

Pathway enrichment analysis further indicated that ILF3^+^ erythroid cells possess stronger proliferative, biosynthetic, DNA repair, and heme metabolic capacities, while ILF3^−^ cells are dominated by inflammatory and stress responses. These results suggest that ILF3 maintains erythroid metabolic homeostasis by repressing stress-related pathways.

Columns represent results from AUCell, UCell, singscore and ssGSEA. Asterisks indicate statistical significance determined by RRA (*P < 0.05, **P < 0.01, ***P < 0.001). (B) Bar plots showing the concordance of pathway directionality across AUCell, UCell, singscore, and ssGSEA methods.

### Successful Construction and Transfection of Lentiviral Vectors

**Fig 7:**
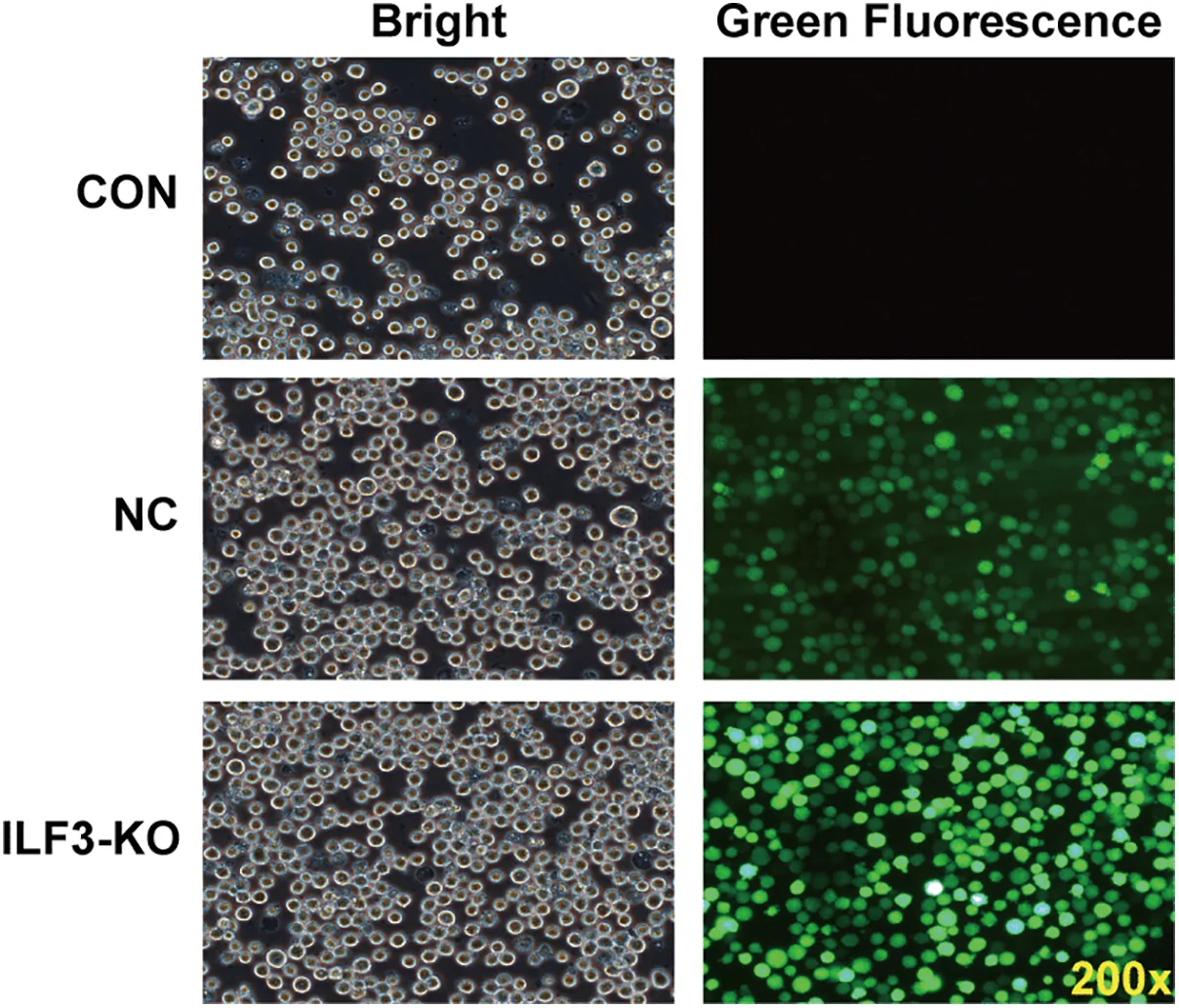
Fluorescence microscopy images of K562 cells after 72h transfection in each group. CON, normal target K562 cells; NC, K562 cells infected with negative control virus; ILF3-KO, K562 cells infected with LV-ILF3-sgRNA (09814−1).

Polymerase chain reaction (PCR) and Sanger sequencing confirmed the successful construction of the ILF3 knockout (ILF3-KO) lentiviral vector. Fig 7 shows fluorescence microscopy images of cells in each group at 72 h after viral transfection. Under bright-field microscopy, K562 cells with normal morphology were observed in the blank control group (CON), negative control group (NC), and ILF3 knockout group (ILF3-KO). Under green fluorescence microscopy, strong green fluorescent signals were observed in the NC and ILF3-KO groups, with more than 80% of cells expressing green fluorescent protein (GFP), indicating successful viral transfection. No green fluorescent signal was detected in the CON group.

### Effects of ILF3 Knockout on Proliferation and Apoptosis of K562 Cells

**Fig 8:**
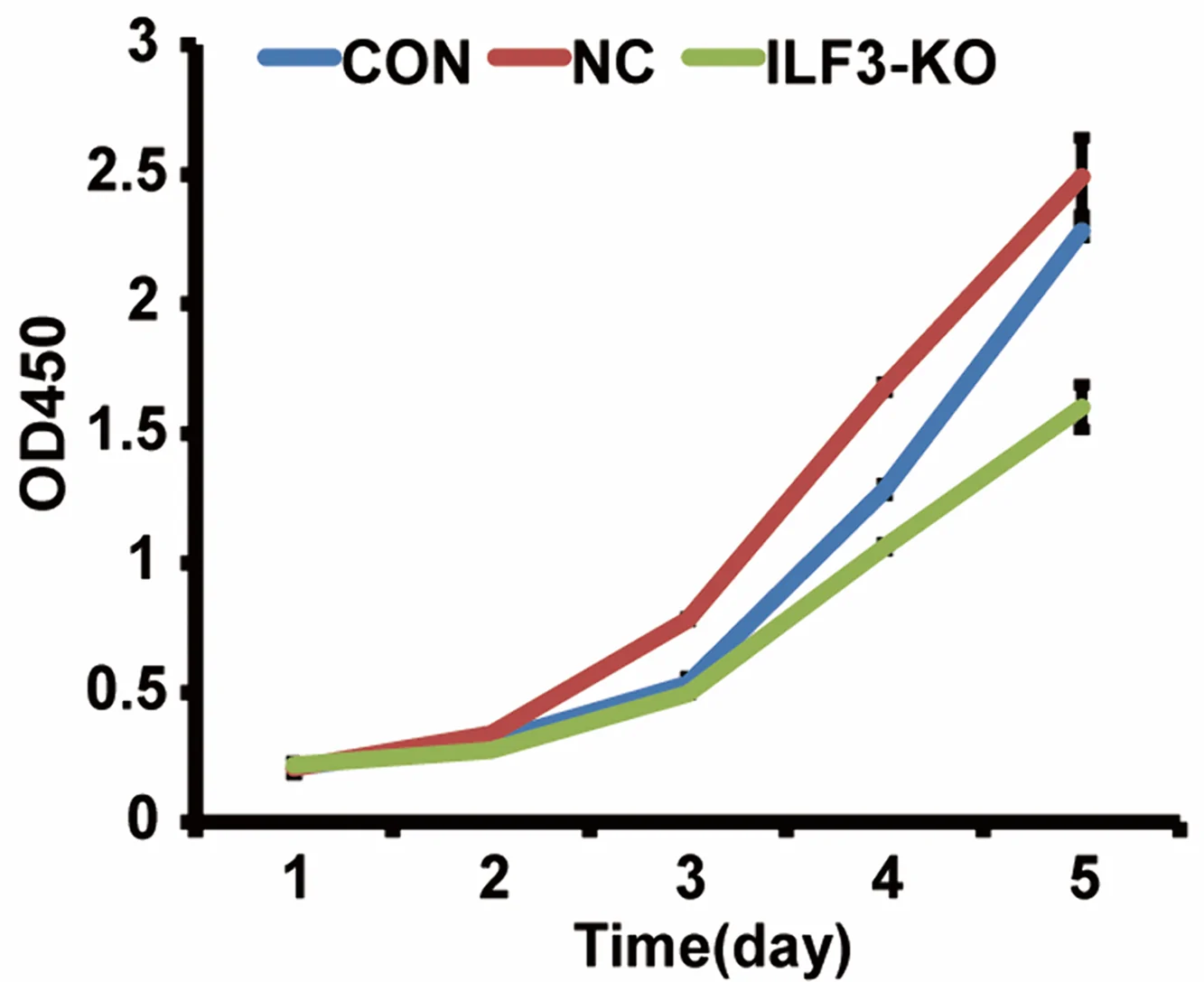
Effect of ILF3 knockout on K562 cell proliferation. Changes of 450nm light absorbance of K-562 cells after knocking out ILF3 (NC VS ILF3-KO: P < 0.05).

CCK-8 assay results showed that on day 5 after infection, the absorbance value was 1.602 ± 0.087 in the ILF3-KO group and 2.491 ± 0.149 in the NC group (S1 Data). The value in the ILF3-KO group was significantly lower than that in the NC group (P < 0.001) (Fig 8 and S1 Fig). Furthermore, the difference between the two groups exceeded 20%, indicating that ILF3 knockout inhibited the proliferation of K562 cells.

**Fig 9:**
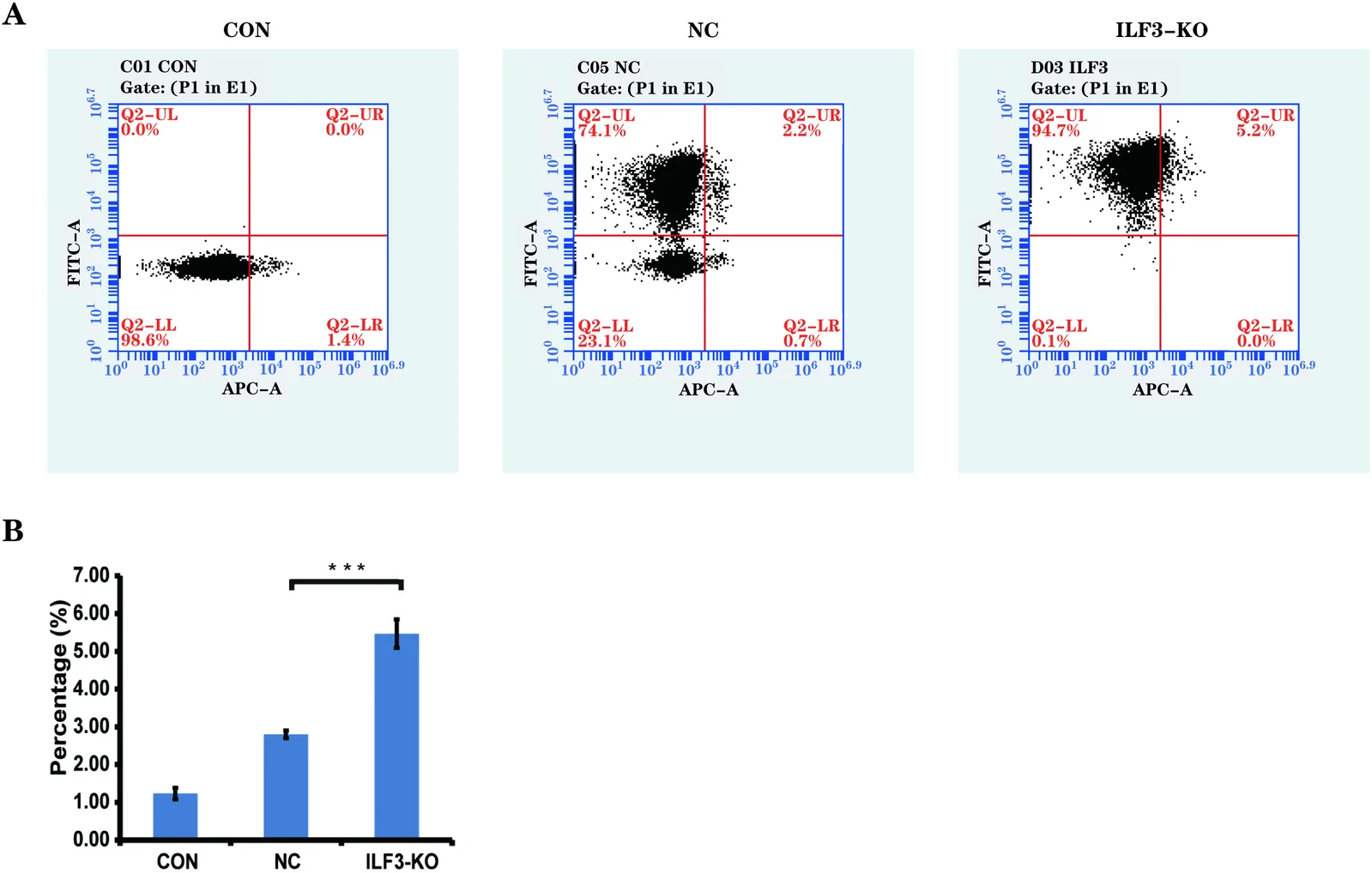
Effect of ILF3 knockout on K562 cell apoptosis. (A) The results of flow cytometry. (B) Comparison of cell apoptosis rate after knocking out ILF3 (NC VS ILF3-KO: P < 0.05).

Annexin V-APC single staining combined with flow cytometry demonstrated that on day 7 after infection, the apoptosis rates of ILF3-KO, NC, and CON groups were 5.47 ± 0.38%, 2.80 ± 0.10%, and 1.23 ± 0.15%, respectively. Statistical analysis revealed that the apoptosis rate in the ILF3-KO group was significantly higher than that in the NC group (P < 0.001) (Fig 9), with a difference of more than 20%, suggesting that ILF3 knockout promoted apoptosis of K562 cells

## Discussion

By integrating single-cell transcriptomic analysis and in vitro functional validation, this study systematically investigated the expression pattern and potential biological function of ILF3 in erythroid differentiation of patients with β-thalassemia. The main findings are as follows: 1) ILF3 expression was overall downregulated in hematopoietic cells from β-thalassemia patients. 2) ILF3 expression was highly associated with erythroid cells. ILF3^+^ erythroid cells exhibited a mature and metabolically active phenotype, whereas ILF3^−^ cells were enriched with stress and inflammatory signatures. 3) Differentiation trajectory analysis revealed an early-stage arrest in erythroid differentiation in β-thalassemia patients, which was accompanied by low ILF3 expression. 4) In vitro ILF3 knockout significantly inhibited proliferation and promoted apoptosis in K562 cells. These results suggest that downregulation of ILF3 may participate in the pathological process of ineffective hematopoiesis in β-thalassemia by disrupting erythroid metabolic homeostasis, exacerbating inflammatory stress responses, and inducing cell apoptosis.

ILF3 (also known as Nuclear factor 90/110, NF90/NF110) is encoded by the ILF3 gene located on human chromosome 19 and belongs to the family of double-stranded DNA- and RNA-binding proteins (DRBPs). Recent studies have increasingly revealed the crucial role of RNA-binding proteins in hematopoietic lineage fate determination. For instance, the RNA-binding protein RBM38 has been reported as a key regulator of erythroid differentiation, which modulates heme biosynthesis by finely regulating the alternative RNA splicing, stability, and translation of Fech, the gene encoding the porphyrin metabolic enzyme ferrochelatase [23]. The present study found that ILF3 was specifically enriched in erythroid cells, and its expression level increased with differentiation progression, suggesting that ILF3 may act as a positive regulator of erythroid maturation. This is consistent with previous studies in embryonic stem cells, in which ILF3 and its complex members participate in the maintenance of pluripotency and coordinate the differentiation process [10]. However, under the pathological context of β-thalassemia, ILF3 expression was systematically downregulated and associated with early arrest of erythroid differentiation, indicating that loss of ILF3 function may disrupt the transcriptional and metabolic programs essential for erythroid differentiation. Notably, this aligns with the observation by several researchers that erythroid differentiation blockade may be related to dysregulated transcription factors. For example, the GATA2-L359V mutation has been found to impair hematopoietic differentiation in mouse embryos and adults [24] and lead to differentiation arrest in chronic myeloid leukemia cells. Our study suggests that ILF3 may also represent a key component of this dysregulated network.

Comparative transcriptomic analysis of ILF3^+^ and ILF3^−^ erythroid cells further revealed its underlying mechanism. ILF3^+^ cells highly expressed mature erythroid marker genes (such as HBB, HBA1/2, ALAS2) and the key regulator KLF1, and were enriched in pathways including cell cycle, DNA repair, oxidative phosphorylation, and heme metabolism. These findings are consistent with the view proposed by Martin-Rufino et al. [4] that transcription factor networks coordinately regulate the inheritance of blood cell traits, suggesting that ILF3 may drive erythroid terminal differentiation and hemoglobin synthesis by promoting erythroid-specific gene expression and maintaining cellular metabolic activity, thus serving a protective role in hereditary anemia diseases. In contrast, ILF3^−^ cells highly expressed stress-related genes (such as BACH1) and fetal hemoglobin repressors (BCL11A, ZBTB7A), and exhibited significant activation of interferon response, TNF-α/NF-κB signaling, and inflammation-related pathways. It is known that the accumulation of free α-globin chains in β-thalassemia triggers oxidative stress and inflammatory responses, leading to increased apoptosis and differentiation arrest in erythroid precursors [1,2]. Our results support the current understanding that an inflammatory microenvironment drives ineffective hematopoiesis [25], indicating that loss of ILF3 may exacerbate this pathological stress state.

ILF3 has also been demonstrated to play a pivotal role in regulating cell proliferation. Previous studies have reported that in K562 cells, ILF3 maintains cellular proliferation by binding to the promoter regions of proliferation-related genes, such as MYC and EGR1, and activating their transcription [11]. In gastric cancer cells, ILF3 promotes cell cycle progression by activating the PI3K/AKT/mTOR signaling pathway. Knockdown of ILF3 was shown to increase the proportion of cells in the G0/G1 phase by 23.6% and decrease the proportion in the G2/M phase by 18.9% [26]. Furthermore, ILF3 is involved in regulating the phenotypic switching of vascular smooth muscle cells. In an atherosclerosis model, ILF3 binds to HMGB1 mRNA to maintain its stability, thereby activating the STAT3 signaling pathway and promoting cell proliferation and migration [27]. Our functional assays in K562 cells revealed that ILF3 knockout significantly impaired cell proliferation and markedly increased the apoptosis rate. As an erythroid leukemia cell line that retains partial erythroid differentiation potential, K562 cells support the notion that ILF3 plays an important role in maintaining the survival and proliferation of erythroid cells. This finding is consistent with studies on the role of the NF90/NF110 complex in maintaining cellular homeostasis [10]. Combined with single-cell sequencing data, we speculate that in the bone marrow microenvironment of β-thalassemia, low expression of ILF3 renders erythroid precursor cells more vulnerable to endogenous stress and inflammatory signals, thereby triggering the apoptotic program and ultimately leading to ineffective hematopoiesis. This is similar to the pathological model in which unpaired α-globin chain toxicity increases the fragility of erythroid precursor cells [2].

This study has several limitations. First, the single-cell data analysis was based on a public dataset with a relatively small sample size. Future studies with expanded patient cohorts are needed to verify the universality of the conclusions. Second, although the K562 cell line is widely used in erythroid differentiation research, it still differs from erythroid differentiation under normal physiological conditions. Further investigations are warranted to validate the functional role of ILF3 in primary CD34^+^ hematopoietic stem/progenitor cell-derived erythroid differentiation models. In addition, the specific molecular mechanisms by which ILF3 regulates erythroid differentiation, such as its target mRNA network, interactions with key erythroid transcription factors (e.g., HBB, KLF1, BACH1, BCL11A, et al), and whether it affects the phenotype of β-thalassemia by regulating γ-globin gene expression, warrant further investigation.

In summary, this study is the first to reveal the important role of ILF3 in erythroid differentiation in β-thalassemia at the single-cell level. Downregulation of ILF3 may contribute to erythroid differentiation arrest and ineffective hematopoiesis by inducing inflammatory stress and apoptosis. ILF3 and its related pathways are expected to serve as novel targets for intervening in erythroid developmental disorders in β-thalassemia.

## Supporting information

S1 File The sgRNA sequences targeting ILF3. (ZIP)

S1 Data Raw OD450 and OD450/fold values. (XLSX)

S1 Fig Effect of ILF3 knockout on K562 cell proliferation. Multiple changes of absorbance at 450 nm of K-562 cells after knocking out ILF3 (NC VS ILF3-KO: P<0.05).
(TIF)
